# Syntaxin 3 is haplosufficient for long-term photoreceptor survival in the mouse retina

**DOI:** 10.3389/fopht.2023.1208805

**Published:** 2023-06-14

**Authors:** Mariajose Perez-Hurtado, Calvin Dao, Amanda E. Saenz, Ruth Heidelberger

**Affiliations:** ^1^ Department of Neurobiology and Anatomy, McGovern Medical School at The University of Texas Health Science Center at Houston (UTHealth Houston), Houston, TX, United States; ^2^ The University of Texas MD Anderson Cancer Center, UT Health Houston Graduate School of Biomedical Sciences, Houston, TX, United States

**Keywords:** STX3, syntaxin 3B, SNAREopathy, retinal degeneration, retinal dystrophy, EOSRD, age-related, ribbon synapse

## Abstract

Biallelic loss-of-function mutations in the syntaxin 3 gene have been linked to a severe retinal dystrophy in humans that presents in early childhood. In mouse models, biallelic inactivation of the syntaxin 3 gene in photoreceptors rapidly leads to their death. What is not known is whether a monoallelic syntaxin 3 loss-of-function mutation might cause photoreceptor loss with advancing age. To address this question, we compared the outer nuclear layer of older adult mice (≈ 20 months of age) that were heterozygous for syntaxin 3 with those of similarly-aged control mice. We found that the photoreceptor layer maintains its thickness in mice that are heterozygous for syntaxin 3 relative to controls and that photoreceptor somatic counts are comparable. In addition, dendritic sprouting of the rod bipolar cell dendrites into the outer nuclear layer, which occurs following the loss of functional rod targets, was similar between genotypes. Thus, syntaxin 3 appears to be haplosufficient for photoreceptor survival, even with advancing age.

## Introduction

Syntaxin 3 is a N-ethylmaleimide-sensitive factor attachment protein receptor (SNARE) protein that catalyzes fusion between vesicles and their target membranes (1). In the retina, syntaxin 3 is expressed exclusively by photoreceptors and bipolar cells (2–5), where it is required for neurotransmitter release (4, 6, 7). Recently, biallelic loss-of-function mutations in the human retinal-specific syntaxin 3 spliceform, syntaxin 3B (*STX3B*), have been linked to an early-onset severe retinal dystrophy in young children (8). Furthermore, biallelic postnatal inactivation of the syntaxin 3 gene (*Stx3*) in mouse photoreceptors has shown to result in the rapid degeneration of photoreceptors and a dramatic thinning of the outer nuclear layer (ONL) of the retina (8, 9). Thus, in addition to catalyzing the exocytic release of neurotransmitter release that underlies chemical synaptic transmission at photoreceptor and bipolar cell synaptic terminals (4, 6, 7), *Stx3* also has an essential role that is necessary for photoreceptor survival.

A cellular hallmark of many inherited disorders of vision is the progressive loss of photoreceptors with age. Given the rapidly devastating consequences of biallelic *STX3/stx3* loss-of-function in both humans and mice, we wondered whether monoallelic *Stx3* loss-of-function might lead to retinal degeneration later in life. As a first step towards addressing this possibility, we examined the outer nuclear layer of older adult mice that were heterozygous for *Stx3* with those of age-matched controls. Analysis of outer nuclear layer thickness, number of photoreceptor somata and sprouting of bipolar cell dendrites into the outer nuclear layer (ONL) indicated that inactivation of a single allele of *Stx3* does not drive age-related photoreceptor loss in the mouse retina.

## Materials and methods

### Animals

Animal procedures conformed to National Institutes of Health guidelines and were approved by the Animal Welfare Committee of the University of Texas Health Science Center at Houston. Male and female mice globally heterozygous for *Stx3* (e.g. *Stx3^+/-^* and *Stx3^f/-^*) and control mice (e.g. *Stx3^wt^*, *Stx3^f/f^*, *Stx3^f/+^*) with a C57Bl6/J background were obtained by the breeding of mouse lines that we developed and characterized previously (8, 10). In contrast to the embryonic lethality of global *Stx3* inactivation in mice, mice that are heterozygous for *Stx3* are viable and fertile (10). Genotyping was performed by PCR using DNA isolated from tail snips (10, 11) and independently confirmed at least once. Founder lines were negative for the retinal degeneration mutations *Rd1* and *Rd8* (8). Mice were kept under standard housing conditions with unlimited access to food and water and with a 12 h light/dark cycle and euthanized by cervical dislocation followed by decapitation at 14-26 months of age. The mean age and age range was comparable between groups (control: 22 ± 1 months (range 14-26 months), n=15; *Stx3* het: 20 ± 2 months (range 17-26 months), n=7; p = 0.6922).

### Tissue preparation and immunolabeling

Following euthanasia, eyes were enucleated and lenses removed. The eyes were fixed in 4% para-formaldehyde in 0.1M sodium phosphate buffer (RT, 1h). After fixation, eyes were rinsed and cryoprotected in 30% sucrose (PBS, overnight, 4°C), embedded in OCT embedding medium (Tissue-Tek, Torrance, CA), fast-frozen, and sectioned into 14-16 µm cryostat sections. Sections from the central retina were collected on Superfrost Plus Gold microscope slides (Fisherbrand, Pittsburgh, PA) and stored at − 20°C until use. For immunolabeling, sections were thawed and incubated in blocking solution (5% normal donkey serum and 0.3% Triton X-100 in PBS) for 1 h, and primary antibodies were applied overnight at room temperature. After washing, secondary antibodies were applied for 2 h at room temperature. Sections were rinsed and cover-slipped in ProLong Gold antifade mounting medium with DAPI (Invitrogen, Eugene, OR). The retinal distribution of Stx3B was visualized with monoclonal antibody 12E5 raised against stx3 (MilliporeSigma, Burlington, MA, United States) (12), which we characterized further in Campbell et al., 2020, Supplementary Figure 1 (13). Rabbit monoclonal PKC alpha (ab32376, Abcam, Cambridge, UK) was used to label rod bipolar cells and their dendritic processes (14, 15). Secondary Cy3 conjugated donkey anti-mouse IgG (MilliporeSigma, Burlington, MA, United States), and Alexa Fluor 488 donkey anti-rabbit IgG (Jackson ImmunoResearch, West Grove, PA) were used for visualization. All antibodies were used at a 1:200 dilution.

### Imaging and image analysis

Image acquisition and data analysis were conducted in similar manner to that described previously (13). Rod spherules and cone pedicles in retinal sections were identified by their characteristic appearance and respective locations within the outer plexiform layer (OPL) and by immunolabeling for Stx3 (2, 3, 13, 16). Images (Z-stacks) were acquired on a Zeiss 800 confocal microscope (Carl Zeiss Microscopy GmbH, Oberkochen, Germany). Analysis of images was performed blinded to genotype. Measurement of outer nuclear layer thickness and photoreceptor somata number was performed in ImageJ (17). The thickness (μm) of the outer nuclear layer (ONL) was measured in maximum intensity projections using the Image J straight tool. Photoreceptor nuclei were quantified in an 800 µm^2^ region of the ONL using the “grid” function of Image J and counted using the “multi-point” tool. Measurement of rod bipolar cell dendrite length was performed in Fiji/ImageJ2 (17, 18). Dendritic lengths were calculated in maximum intensity projections using the free hand line tool. Dendrites were traced from the border between the outer plexiform layer and outer nuclear layer to their terminal ends in the outer nuclear layer. For each measure, 1-4 histological sections were analyzed per mouse and results averaged together to produce a single value per animal for each measure. Data were compiled in Excel (Microsoft, Redmond, WA, United States), and statistical analyses were performed in Prism 7 (GraphPad Software, Inc., San Diego, CA, United States) using the Mann Whitney Test. Figure images are displayed as maximum intensity projections. Results are represented as mean ± SEM, where “n” represents the number of mice.

## Results

**Figure 1:**
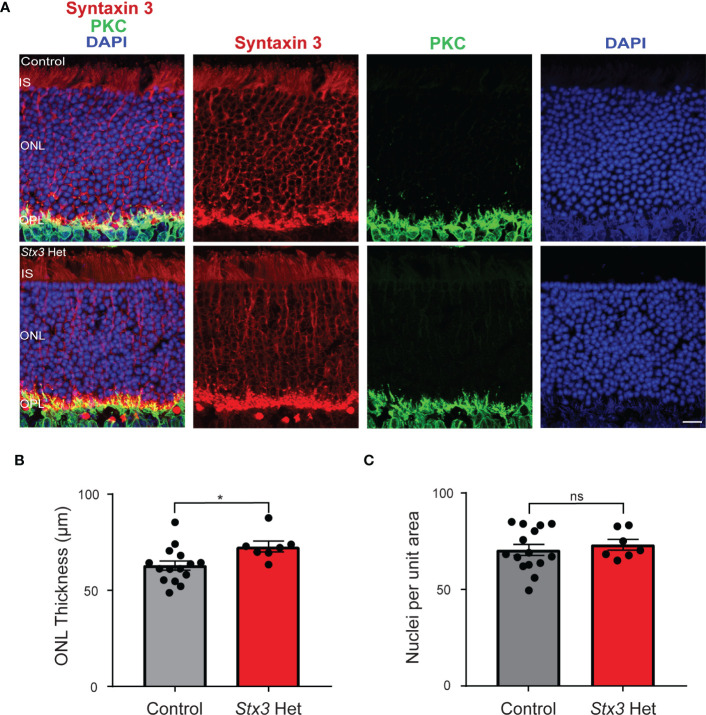
Outer nuclear layer (ONL) thickness and photoreceptor number are not reduced by *Stx3* heterozygosity in the aged mouse retina. **(A)** A representative pair of confocal images from an older adult mouse heterozygous for *Stx3* and an older adult control mouse show that the ONL was preserved in the *Stx3* het mouse and comparable to that of the control. Stx3 (red), PKC (green), and DAPI (nuclear marker, blue). Scale bar 10µm. IS Inner segments, ONL outer nuclear layer, OPL outer plexiform layer. **(B)** The average thickness of the ONL was similar between groups, although it was slightly larger in the *Stx3* het mice (p: 0.0164). **(C)** The number of nuclei in an 800 µm^2^ area of the ONL was not different between groups (p: 0.6173). For **(B, C)**, *Stx3* hets, n= 7 mice and for controls, n=15 mice. * denotes p value <0.05 and ns denotes p value is not significant.

In this study, we asked whether having only a single functional *Stx3* allele might be a risk factor for age-related photoreceptor death. To address this question, we examined and compared the retinae of older adult mice that were heterozygous for *Stx3* with those of similarly-aged controls (control: 22 ± 1 months, n=15; *Stx3* het: 20 ± 2 months, n=7; p = 0.6922). In the representative confocal images shown in **Figure 1A** and in the compiled data from multiple animals (**Figure 1B**), ONL thickness was not diminished in mice that were heterozygous for *Stx3* when compared to controls (control: 63 ± 2 µm, n=15; *Stx3* het: 73 ± 3 µm, n=7; p = 0.0164). Furthermore, there was no difference in the number of photoreceptor somata per unit area between groups (**Figures 1A, C**; control: 70.5 ± 2.9, n=15; *Stx3* het: 73.2 ± 2.8, n=7; p = 0.6173). Together, these results indicate that the outer nuclear layer (ONL) in mice heterozygous for *Stx3* is comparable to that of control mice (**Figure 1**).

**Figure 2:**
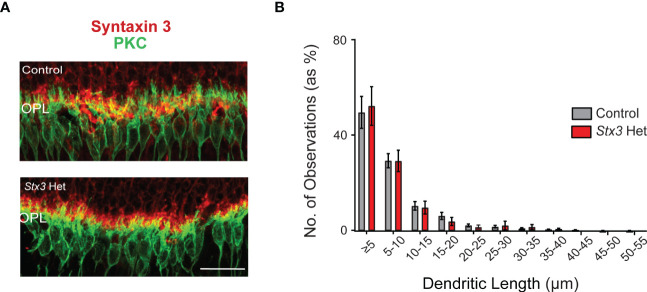
Rod bipolar cell dendritic length is not altered by *Stx3* heterozygosity in the older adult mouse retina. **(A)** A representative pair of confocal images through the outer plexiform layer (OPL) of an older adult heterozygous *Stx3* mouse and an older adult control mouse were labeled with antibodies against Stx3 (red) and PKC (green), show similar dendritic lengths. Scale bar 20 µm. OPL outer plexiform layer. **(B)** The amplitude distributions (by percentage) of dendritic lengths of rod bipolar cells in older adult mice heterozygous for Stx3 and aged control mice were virtually identical and not statistically different. *Stx3* hets, n= 7 mice and for controls, n=15 mice.

When rod photoreceptors die and/or their ribbon-style synapses become non-functional, rod bipolar cells extend their dendrites beyond the outer plexiform layer (OPL) and into the ONL (19–23). We therefore measured and compared the length of PKC-labeled rod bipolar cell dendrites as a proxy of rod photoreceptor loss in older adult mice heterozygous for *Stx3* and in similarly-aged control mice. Results show that dendritic lengths were virtually identical amongst the two groups, with each group having a similar percentage of dendritic length distributions that included the occasional longer ONL sprout (**Figure 2**). Taken together, these results demonstrate that one functional *Stx3* allele is sufficient to maintain long-term photoreceptor viability.

## Discussion

Syntaxin 3 is the only plasma membrane syntaxin known to be expressed by photoreceptors (2, 3, 24). In humans, biallelic loss-of-function mutations in syntaxin 3A, a syntaxin 3 splice form expressed widely throughout the body outside of the retina, gives rise to a devastating gastrointestinal disorder that presents in infancy (8, 25, 26). If the mutations are located in exons that are conserved between syntaxin 3A and the retinal-specific syntaxin 3 spliceform, syntaxin 3B (5, 8), the children additionally exhibited an early onset severe retinal dystrophy (8). In mice, the global inactivation of *Stx3* is embryonic lethal (10), while inactivation of *Stx3* selectively in photoreceptors produced a rapid loss of photoreceptors and a dramatic reduction in ONL thickness (8, 9). Thus, in addition to its role in synaptic transmission at retinal ribbon-style synapses (4, 6, 7), syntaxin 3 is also required for photoreceptor survival.

In this study, we examined the effects of deletion of a single *Stx3* allele on the outer retina. We found no difference in the thickness of the outer nuclear layer between older adult controls and older adult mice that were heterozygous for *Stx3*. In addition, we did not observe a decrease in the number of photoreceptor somata per unit measure or an increase in the sprouting of rod bipolar cell dendrites. The latter might be expected if rods had died or retracted their spherules at a higher rate in older adult *Stx3* heterozygous mice relative to age-matched controls or if the *Stx3* heterozygous rod to rod bipolar cell synapses were non-functional (19, 21–23). We did note that most of the rod bipolar cell dendrites in older adult *Stx3* heterozygous mice appropriately contacted rod terminals, suggesting that the primary reason for a lack of exuberant dendritic sprouting is that the dendritic targets, the rod terminals, demarcated by Stx3 immunolabeling, were still present and located close to the OPL/ONL border.

One of the motivations for conducting this study was to predict whether loss-of-function mutations in one *STX3* allele might increase the risk of photoreceptor loss in human subjects later in life. Our results suggest that *Stx3* is haplosufficient for photoreceptor survival, even at older ages. However, the situation could be very different if, rather than a loss-of-function mutation, there were a monoallelic dominant negative mutation. Indeed, SNAREopathies have been reported in which dominant mutations in one gene negatively affect the functionality of the wild-type transcript (27). Interestingly, of the identified human *STX3* mutations associated with visual impairment to-date, all have been biallelic loss-of-function mutations (8). Thus, for these patients, the introduction of a wild-type gene could be sufficient to rescue the remaining photoreceptors and prevent further photoreceptor loss.

## Data availability statement

The raw data supporting the conclusions of this article will be made available by the authors, without undue reservation.

## Ethics statement

The animal study was reviewed and approved by Animal Welfare Committee of the University of Texas Health Science Center at Houston.

## Author contributions

RH conceived and directed the project. MP-H and AS performed the experiments. MP-H, CD, and RH analyzed data. All authors contributed to data interpretation. RH and MH-P prepared the first draft. All authors contributed to the article and approved the submitted version.
